# Balancing Rigidity and Safety of Pedicle Screw Fixation via a Novel Expansion Mechanism in a Severely Osteoporotic Model

**DOI:** 10.1155/2015/318405

**Published:** 2015-02-01

**Authors:** Thomas M. Shea, James J. Doulgeris, Sabrina A. Gonzalez-Blohm, William E. Lee, Kamran Aghayev, Frank D. Vrionis

**Affiliations:** ^1^Department of Chemical & Biomedical Engineering, University of South Florida, Tampa, FL 33620, USA; ^2^H. Lee Moffitt Cancer Center & Research Institute, Neurooncology Department, Tampa, FL 33612, USA; ^3^Departments of Neurosurgery and Orthopedics, Morsani College of Medicine, University of South Florida, Tampa, FL 33612, USA

## Abstract

Many successful attempts to increase pullout strength of pedicle screws in osteoporotic bone have been accompanied with an increased risk of catastrophic damage to the patient. To avoid this, a single-armed expansive pedicle screw was designed to increase fixation strength while controlling postfailure damage away from the nerves surrounding the pedicle. The screw was then subsequently tested in two severely osteoporotic models: one representing trabecular bone (with and without the presence of polymethylmethacrylate) and the other representing a combination of trabecular and cortical bone. Maximum pullout strength, stiffness, energy to failure, energy to removal, and size of the resulting block damage were statistically compared among conditions. While expandable pedicle screws produced maximum pullout forces less than or comparable to standard screws, they required a higher amount of energy to be fully removed from both models. Furthermore, damage to the cortical layer in the composite test blocks was smaller in all measured directions for tests involving expandable pedicle screws than those involving standard pedicle screws. This indicates that while initial fixation may not differ in the presence of cortical bone, the expandable pedicle screw offers an increased level of postfailure stability and safety to patients awaiting revision surgery.

## 1. Introduction

Osteoporosis is a global public health problem affecting over 200 million people worldwide, with 44 million affected in the United States [1]. Clinicians consider this medical condition a potential risk factor for spinal instrumentation and its incidence, for example, has been reported to be higher than 50% in Korean females undergoing spine surgery [2]. Since life expectancy is increasing and osteoporosis mainly afflicts the geriatric community, investigations estimate that the number of osteoporotic patients in spinal surgery will also increase [3].

Most current procedures in spinal fusion restrict mobility in one or multiple levels of the spine so that, over time, arthrodesis can occur. The current clinical “gold standard” of spinal fusion is the bilateral pedicle screw system [4]. However, since a reduction in bone quality leads to nonunion, screw pullout, and other complications, adequate fixation within the bone-screw interface for patients with osteoporosis represents a continuous challenge.

To overcome this issue, some researchers have considered a variety of alterations to screw design or insertion technique to increase the amount of fixation in low density bone. However, many of the more successful techniques also involve an increased level of risk to the patient. For instance, increasing the diameter of a pedicle screw has been shown to increase the purchase between thread and bone [5–7], but pedicular fracture may occur when being instrumented in low density bone [8, 9]. Expandable pedicle screws [10–12] and anchors [13, 14] can significantly increase fixation in bone of a compromised quality while keeping the screw diameter within the pedicle at a safe size; however expansion medial or inferior to the pedicle could result in damage to the spinal cord [15, 16] or nerve roots [17] should failure occur. Bicortical fixation has the potential to more than double the pullout force in osteoporotic bone [7], but improper placement may result in puncture of the aorta or iliac vessels on the anterior side of the vertebral body [8, 18]. Additionally, while polymethylmethacrylate (PMMA) augmentation produces some of the most significant increases in pullout force over standard pedicle screws alone [12, 19–23], its high polymerization temperatures [24–26], potential for leakage into the vertebral canal [19, 21, 23, 27, 28], inability to degrade over time [29–31], and toxic monomer [14, 32] expose patients to the possibility of further complications.

Investigations often use pullout testing to determine the fixation efficacy of pedicle screws in the human vertebra regardless of bone quality. They are often performed in laboratory conditions on either cadaveric [7, 10, 11, 14, 19] or synthetic [5, 6, 22, 27, 28] models. Synthetic bone models are widely accepted due to their homogeneity and data reproducibility when compared to cadaveric samples [5, 6, 22, 27, 28].

Considering the dangers associated with many of the above techniques, the question arose if it were possible to design a pedicle screw that would increase pullout strength when instrumented in osteoporotic individuals while avoiding catastrophic damage to the patient should failure occur. Therefore, the aim of this investigation was to test a concept of pedicle screw design that limited radial expansion to a single direction and obtain preliminary data on its stability and potential safety through axial pullout tests in polyurethane test blocks. It was believed that the addition of a single expandable arm would not only provide the expandable pedicle screws superior pullout strength over the standard screws but also result in similar measurements of synthetic bone loss between the two screw types in all directions surrounding the screw except for the direction of the expandable arm, which is expected to remove a greater amount of polyurethane foam exclusively in that direction. Clinically, the ability to position the expandable arm in the superolateral direction of the pedicle should theoretically control bone failure in a way that avoids potential catastrophic damage to the nerves surrounding the pedicle.

## 2. Materials and Methods

### 2.1. Instrumentation

**Figure 1:**
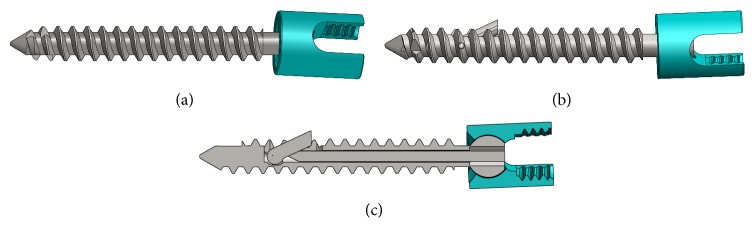
Illustration of pedicle screws used in this study. (a) Standard pedicle screw. (b) Single-armed expandable pedicle screw. (c) Single-armed expandable pedicle screw (sectional view).

Twelve (12) 6.5 × 45 mm Ti-6Al-4V standard pedicle screws were used in this investigation (Figure 1(a)). The titanium alloy used in the current study is commonly found in other spinal implants due to its proven biocompatibility and superior osseointegration to other frequently used materials such as stainless steel [33]. To ensure that no differences in screw diameter, pitch, or thread design existed between test groups, six (6) of these screws were modified to incorporate an expandable arm (Figure 1(b)). These modifications included a slot for which the pinned appendage was to be incorporated as well as a 2.20 ± 0.25 mm diameter cannulation extending along the screw's longitudinal axis to the most distal edge of the slot. Following screw instrumentation, a pin 2.00 ± 0.25 mm in diameter was inserted through the cannulation, allowing for an initial expansion of the appendage of 25° ± 1.5° (Figure 1(c)); however the arm was free to rotate up to 90°. Keeping the pin within the screw following placement of the rod prevented accidental postoperative recession of the appendage back into the screw shaft. The singular expandable arm on the screw was designed so that when used* in vivo*, the arm could be positioned in the superolateral direction to the pedicle. This way, should bone fracture occur with this screw, it was proposed that any additional damage to the vertebral body over what would normally be seen in a standard pedicle screw would be located in an area away from potential harm of the surrounding nerves.

### 2.2. Testing Blocks

**Figure 2:**
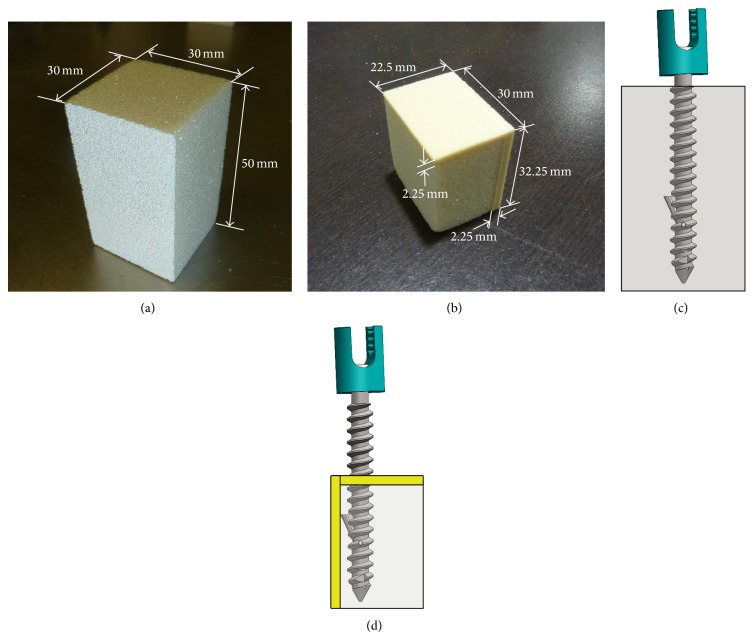
(a) Trabecular and (b) composite testing blocks with a representation of an implanted single-armed expandable screw in (c) trabecular and (d) composite blocks.

Testing blocks were obtained from Sawbones (Pacific Research Laboratories Inc., Vashon Island, WA, USA). Two sets of polyurethane blocks of different sizes and compositions were used in the current study (Figure 2). One, referred to in this study as trabecular block, measured 30 mm × 30 mm × 50 mm and had a density of 0.08 g/cm^3^, a density similar to that of severely osteoporotic trabecular bone. The other block, referred to as composite block, was designed to simulate the interaction of the expandable arm as it makes contact with cortical bone. This block consisted of sections of varying densities to simulate both the trabecular and cortical bone comprising severely osteoporotic vertebral bodies. The portion of the block representing trabecular bone measured 20.25 mm × 30 mm × 30 mm and had a density of 0.08 g/cm^3^. The sections of the block representing the cortical bone were 2.25 ± 0.25 mm thick and had a density of 0.64 g/cm^3^. This higher density portion was layered along the top and one of the sides of the trabecular section. The thinner design of the composite block in comparison to the trabecular is to allow the arm of the pedicle screw to open within the proximity of the cortical wall while permitting the screw to be in the center of the testing machine.

### 2.3. Bone Cement

The PMMA used in this study was provided by DFINE, Inc. (San Jose, CA, USA), and was composed of a powder (made up of 69.5% PMMA, 30.0% barium sulfate, and 0.5% benzoyl peroxide) and liquid monomer (made up of 99.5% methylmethacrylate, 0.5% N-N dimethyl-p-toluidine, and 75 ppm hydroquinone). Approximately 2.0 mL of the cement was injected into the test block prior to screw insertion for each PMMA test. The cement had a working time of thirty minutes. All screws were implanted within this window and then the cement was allowed to fully harden for over 12 hours prior to testing.

### 2.4. Implantation

The pedicle screws were inserted into the test blocks by placing a specially designed cap over the blocks to locate proper placement of the pilot hole and to increase confidence that the screw was inserted perpendicular to the top surface. Markings on the screws indicated not only the proper depth to which the screws were to be inserted (45 mm in the trabecular blocks and 30 mm in the shorter composite blocks) but also the location that the expandable arm was facing inside the foam. All screws were driven up until the marking reached the surface of the test block while ensuring at that point that the expandable arm, if applicable, was positioned in its desired location (directly facing any of the four sides of the trabecular blocks or the cortical side of the composite block). The arms on the expandable screws were expanded after insertion and proper expansion of the arm was confirmed using an OEC 9400 C-arm (GE OEC Medical Systems, Salt Lake City, UT, USA).

### 2.5. Testing Procedure

Pullout testing was performed in six conditions (*n* = 6 for each): (1) standard pedicle screws in trabecular block, (2) expandable pedicle screws in trabecular block, (3) PMMA augmented standard pedicle screws in trabecular block, (4) PMMA augmented expandable pedicle screws in trabecular block, (5) standard pedicle screws in composite block, and (6) expandable pedicle screws in composite block. Test order was randomized among all conditions, except for the ones involving bone cement augmentation (conditions 5 and 6), which were tested last since PMMA injection was considered to create irreversible alterations to the screws.

**Figure 3:**
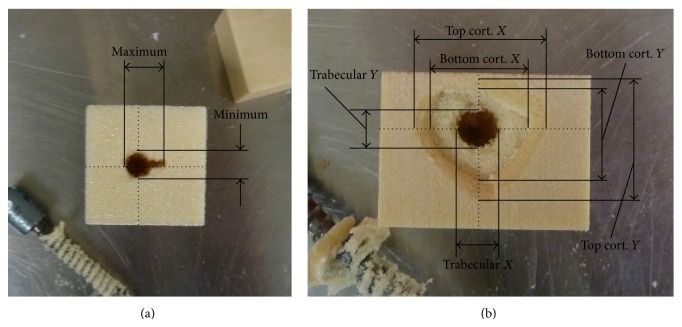
Measurements recorded following pullout in (a) trabecular block and (b) composite block.

The machine used for providing the set displacement and recording the associated axial load was the MTS 858 MiniBionix (MTS Systems Corporation, Eden Prairie, MN, USA). The hydraulic clamp, attached to the base of the machine, kept the pedicle screw stationary during pullout testing. The fixation frame, which was attached to the biaxial load cell, ensured that the test blocks were held stationary relative to the load cell and did not wobble or become displaced in any other manner. The weight of the fixation frame was taken into consideration by zeroing the output values of the load cell prior to testing. Axial displacement occurred at a rate of 5 mm/min and continued until the pedicle screw was completely removed from the test block. At that point, the damage to the test block was measured at each desired level using a set of digital calipers (±0.03 mm) centered at the original location of screw placement and in the directions parallel to the sides of the block, as illustrated in Figure 3. For the trabecular blocks, only the top surface was measured while three levels were measured in the composite blocks: the top and bottom surface of the top cortical layer and the top surface of the trabecular section. For all tests, except for those involving PMMA augmentation, the pullout tests for each screw were repeated up to three times in new test blocks and the resulting values were averaged.

Mechanical parameters recorded during pullout testing included pullout force, stiffness, energy to failure, and energy to removal. Pullout force (N) was defined as the maximum axial load achieved during the entirety of the test. Stiffness (N/mm) was defined as the approximate slope on the load displacement curve leading up to the point of screw pullout. Energy to failure (N∗mm) was defined as the area under the load displacement curve up until pullout was reached. Energy to removal (N∗mm) was defined as the area under the entirety of the load displacement curve.

Statistical analysis was performed using SAS 9.2 (Cary, NC, USA). Using a Shapiro-Wilk test, normality was verified for all measurements and mechanical parameters except for the damage size produced by PMMA augmented screws. Thus, *t*-tests were performed for comparing all values between the standard and expandable pedicle screw conditions. For comparisons between PMMA augmented and nonaugmented screws of the same type, a paired *t*-test was used to determine differences between each of the mechanical parameters while a Wilcoxon signed rank test was used to determine differences between the measurements of damage. A significance level of 0.05 was used for all statistical comparisons. The power for all statistically significant comparisons observed was at or above 80%, unless otherwise noted (in which case, further analysis was performed to determine what sample size would be required to reach this power).

## 3. Results

Due to a malfunction of the C-arm during the testing procedure, six (6) of the blocks instrumented with expandable pedicle screws could not be imaged. Therefore, to determine if the arm was properly expanded, the photographs of all the expandable screws were reevaluated after the pullout testing was performed. If the arm exited the block fully extended, it was determined that the arm was open enough to “catch” the foam block as it was being pulled out and adequate expansion was confirmed. On the other hand, if the arm failed to be pulled open by the surrounding low density foam, it was determined that the arm was not initially opened to an appropriate angle and the results for that individual test were disregarded. There were three (3) such instances of this happening, in which case additional pullout tests in new testing blocks had to be performed.

### 3.1. Mechanical Testing

**Figure 4:**
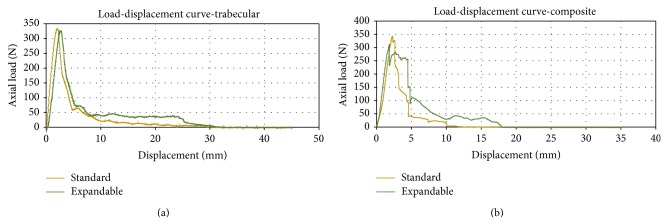
Load-displacement curves comparing individual standard and expandable screw pullout tests in (a) trabecular blocks and (b) composite blocks.

The mean and standard deviation of the values returned for each mechanical parameter can be found in Table 1. An example of load-displacement curve showing individual pullout test results for both a standard and expandable screw in both models of test blocks can be seen in Figure 4.

For tests performed in trabecular blocks, the expandable pedicle screws produced a mean pullout force 5.4% less than standard pedicle screws (*P* < 0.01) while showing no statistical differences in stiffness (*P* = 0.07) or energy to failure (*P* = 0.11) and an increase in energy to removal of 26.3% (*P* < 0.001). PMMA augmentation of the standard pedicle screws produced no noticeable differences in pullout (*P* = 0.98), stiffness (*P* = 0.06), or energy to failure (*P* = 0.43) when being compared to the nonaugmented standard pedicle screw. It did, however, increase the energy to removal of the standard screws by 97.3% (*P* < 0.001). PMMA augmented expandable pedicle screws tested against nonaugmented expandable screws demonstrated an increase in pullout strength of 12.3% (*P* = 0.02; power = 73.22%; suggested sample size = 7), energy to failure of 33.4% (*P* = 0.01), and energy to removal of 94.6% (*P* = 0.001). PMMA augmentation, however, did not produce any statistical differences in stiffness in the expandable pedicle screws (*P* = 0.50).

In the composite blocks, the expandable pedicle screws produced no statistical differences compared to the standard pedicle screws with regard to pullout force (*P* = 0.47), stiffness (*P* = 0.86), and energy to failure (*P* = 0.69). There was, however, a significant increase in the energy to screw removal as the expandable pedicle screws produced mean values 46.3% greater than standard pedicle screws (*P* = 0.001).

### 3.2. Test Block Damage

**Figure 5:**
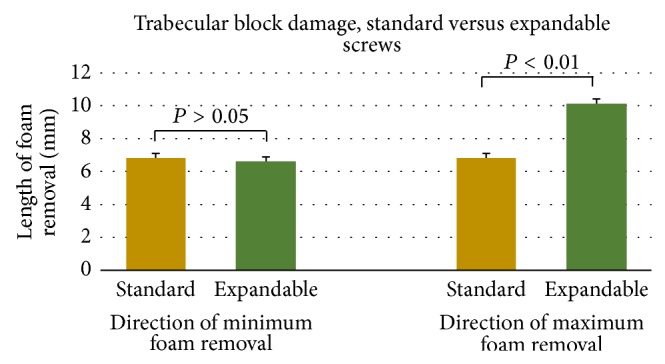
Graphical representation of postpullout damage to trabecular test blocks between nonaugmented standard and expandable pedicle screws. Data is presented as mean and standard deviation.

**Figure 6:**
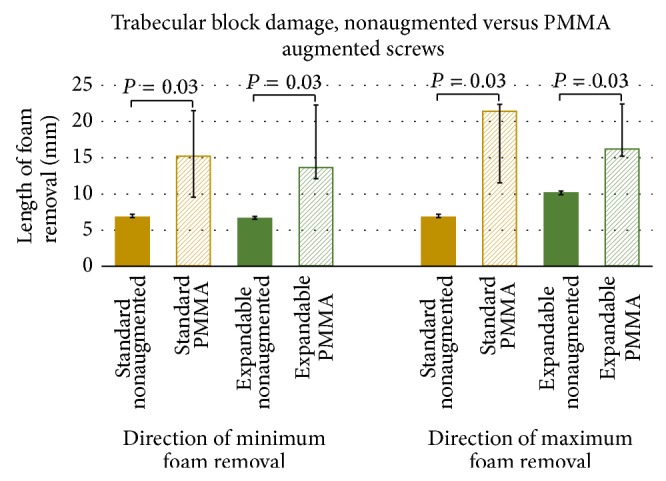
Graphical representation of postpullout damage to trabecular test blocks between nonaugmented and PMMA augmented pedicle screws of the same design. Data here is presented as median and range due to the nonparametric nature of the statistical test performed.

**Figure 7:**
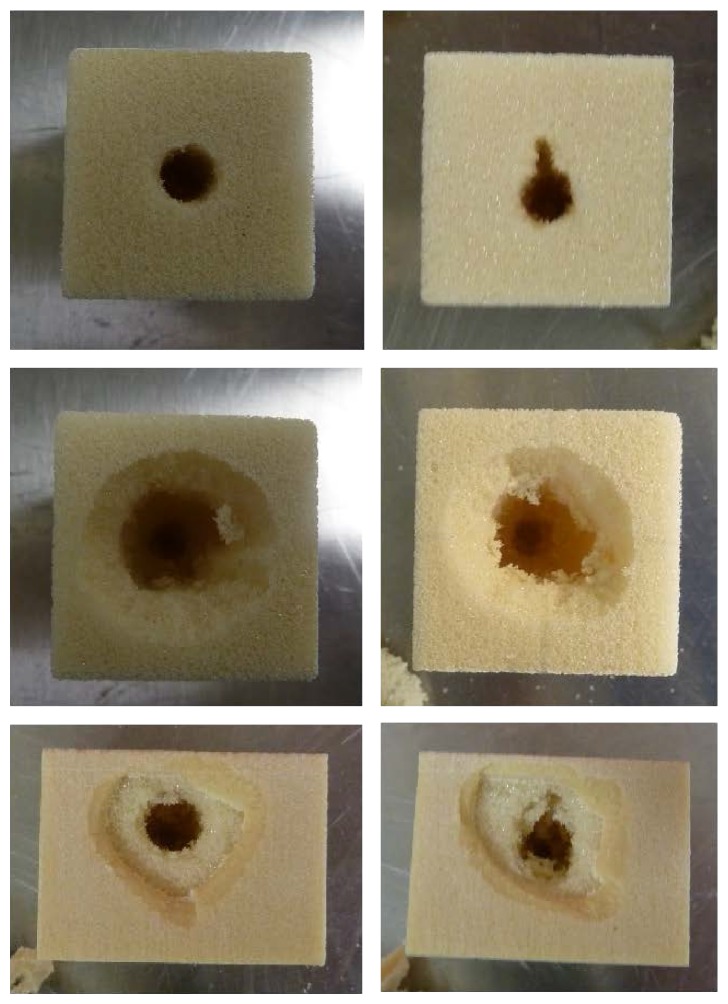
Representation of resulting damage after pullout test of the standard (left) and expandable (right) screws in the nonaugmented trabecular block (top), trabecular block with PMMA augmentation (center), and composite block (bottom).

For the pullout tests performed in trabecular blocks, standard pedicle screws produced cleanly sheared holes approximately the size of the outer diameter of the screw. Similarly, the expandable pedicle screws left cleanly sheared holes caused by the threading of the screw; however an additional slot was present as a result of the expanded arm. PMMA augmentation of both screw types resulted in a large asymmetrical removal of foam, often conical in shape. Graphical representations of polyurethane foam removal following pullout testing in trabecular blocks can be found in Figure 5 (standard versus expandable) and Figure 6 (nonaugmented versus PMMA augmentation). Images of the resulting damage under all six conditions tested can be found in Figure 7.

While the expandable pedicle screw produced similar amounts of foam removal to the standard screw as a result of its outer threading (*P* = 0.06), the addition of the expandable arm increased the distance of block damage by 47.4% (*P* < 0.01). PMMA augmentation of the standard pedicle screws increased block damage by 122% (*P* = 0.03) to 212% (*P* = 0.03) when compared to the nonaugmented standard pedicle screws. Similarly, augmentation of the expandable pedicle screws increased the amount of trabecular block damage by 58.7% in both tests' maximum damage recording (*P* = 0.03) and 104% in the direction of both tests' minimum amount of recorded damage (*P* = 0.03).

**Figure 8:**
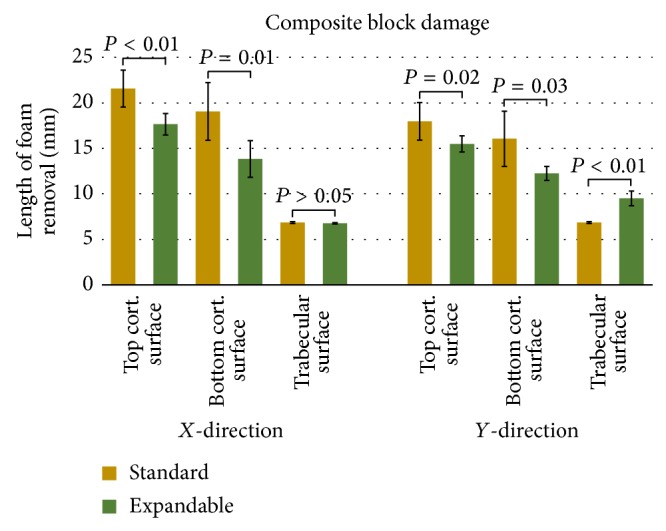
Graphical representation of postpullout damage to composite test blocks between standard and expandable pedicle screws. Data is presented as mean and standard deviation.

Pullout testing in the composite blocks resulted in a more asymmetrical shape of foam removal than was witnessed in the trabecular block. Although not typically as clean of a shear, the trabecular section of the composite blocks similarly presented a hole approximately the size of the outer diameter of the screw with more foam removal in the direction of arm extension for the expandable screw. The top layer of cortical foam, however, was often removed in an irregular, conical shape extending well past the diameter of the screw threads (Figure 7). A graphical representation of polyurethane foam removal following pullout testing in composite blocks can be found in Figure 8.

While the expandable pedicle screws produced a similar amount of low density foam removal as the standard pedicle screws due to the screw's threading (*P* = 0.10), the expandable arms increased the distance of foam removal by 38.7% in that direction (*P* < 0.01). In the top cortical layer, the expandable pedicle screws created significantly less damage in all measured directions than the standard screws. The bottom side of this layer had foam removal averaging 23.7% less in the direction of arm expansion (*P* = 0.03; power = 76.48%; suggested sample size = 7) and 27.4% less in the direction perpendicular to arm expansion (*P* = 0.01) while the top surface averaged 13.8% smaller in the direction of the arm (*P* = 0.02; power = 68.30%; suggested sample size = 8) and 18.1% smaller in the perpendicular direction (*P* < 0.01).

## 4. Discussion

While it is to be acknowledged that pure pullout is not commonly seen in a clinical setting [34], the simplicity and reproducibility of pullout testing make it ideal for comparing screws of different designs or insertion techniques [35]. Besides maximum pullout force, other parameters such as stiffness and the energy being put into the system can provide useful information regarding screw fixation as well. From an engineering standpoint, stiffness describes how much deformation an object sustains given a certain amount of force [36]. Therefore, in the case of a vertebra instrumented with a pedicle screw placed under a given axial load, stiffness indicates not only the displacement of the pedicle screw prior to pullout failure but also how the deformation occurs within the bone-screw interface. The energy placed into the system simulates how much screw displacement will occur through daily activity being performed by the patient. Even after failure occurs, the construct continues to absorb energy as external loads are being applied to it. Therefore, there is an importance placed on how much energy is required not only to reach the point of pullout but also to displace the screw beyond initial failure.

While the expandable pedicle screw performed comparable to the standard screw with regard to pullout in the composite model, the results observed in the trabecular block were less than we anticipated. We hypothesize that this can be attributed to the displacement of foam towards the distal end of the screw as the arm is expanded. This displacement creates a cavity in the direction of pullout resulting in a reduction of both initial contact and initial pullout strength. Alternatively, while this void is still present in the composite test block, the expandable arm is in contact with the side cortical wall, contributing to the screw's resistance to pullout in these models.

In the PMMA model, the equivalent performance of the standard screw with and without PMMA augmentation is contradictory to what is found in the majority of the literature available for both cadaveric [20, 21, 37–39] and synthetic [12, 27, 40] models. Becker et al. [19] reported no significant difference in the pullout strength when augmenting a standard pedicle screw with 2.0 mL of PMMA using a kyphoplasty balloon technique. However, when they injected the same amount of cement without first creating a void, they found a significant increase in pullout strength. Additionally, Paré et al. [23] only reported insignificant differences with PMMA augmentation when using a volume of 0.5 mL in a thoracic model, but more than tripled pullout strength when using 2.0 mL in the lumbar vertebra. Other studies augmenting pedicle screws with 3.0 mL PMMA in low density polyurethane test blocks [27, 40] produced, in some cases, similar values of pullout to what was observed in our study; however their nonaugmented screws failed at forces low enough to produce significant increases through cement usage.

The presence of the side cortical wall proved to play a significant role in fixation of the expandable screw following the incidence of pullout failure, as can be observed by the more gradual decrease in axial load as compared to that of the standard screws (Figure 4). This trend indicates more energy being required to be put into the system to fully remove the screw. Since the only difference between the two designs is that the expandable arm is making contact with the higher density foam, it is suggested that the friction between the wall and the arm is primarily responsible for these higher forces. However, it is difficult to tell if this observation is actually a result of the arm making contact with the thin layer of glue (<0.30 mm thick) holding the sections of the block together. A secondary source of the larger forces is believed to come from the lower density foam interacting with the arm, as can be deduced from the load-displacement diagram for the trabecular block (Figure 4).

To the best of our knowledge, based on our review of the literature, few studies have focused on the amount of energy being placed into the system after initial pullout was reached. Hilibrand et al. [41] reported postfailure energy between standalone pedicle screws and pedicle screws used in conjunction with supralaminar hooks during pullout testing in cadaveric vertebrae. They measured this value from the point of pullout until the device reached a displacement of 5.0 mm from its original position. Somewhat similarly, Zdero and Schemitsch [42] recorded removal energy of surgical screws from the point of maximum force until the force returned to zero. Since pullout may not necessarily occur at the same displacement between tests, we feared this could potentially result in uneven comparisons. Therefore, for the current study, we defined “energy to removal” as the energy required to displace the screw from initial instrumentation to complete extrusion from the block.

In addition to increasing the overall strength of the screw, a main constraint and goal of the expandable screw design was to create a method of controlled failure to reduce the risk of neural injury in a clinical setting. Medial and inferior to the pedicle lie the spinal cord and nerve roots, respectively. They are susceptible to damage as a result of PMMA leakage outside the vertebral body. Additionally, concern may exist that pedicle screws with bilateral or multilateral expansion mechanisms could increase the risk of neural damage due to the increased screw diameter medial and/or inferior to the pedicle. Therefore, the pedicle screw designed for the current study was limited to unilateral expansion intended to be positioned in the superolateral direction to the pedicle. Supporting the objective of controlled failure, the only difference in trabecular block damage between the normal and expanded screw was an additional slot where the arm removed material. Furthermore, when making the same comparison in the composite test block, not only did the expandable screw succeed in creating the desired controlled failure in the trabecular portion, but also it resulted in significantly less damage in all measured directions in the cortical surface. Conversely, the PMMA augmented screws in trabecular block produced a larger and uncontrolled foam removal more than doubled in size of the nonaugmented screws in almost every measured direction (other than that of the nonaugmented expanded arm). This shows the balance between safety and strength of this design.

It is to be acknowledged that limitations to this study are present. Largely due to the high cost of pedicle screws, six standard screws and six expandable screws were used allowing for a maximum sample size of six for all test conditions. However, to enhance reproducibility of the results, homogeneous synthetic bone models were used in place of cadaveric human vertebra of varying bone mineral densities.

An additional limitation lies in the composite block. While it was designed to serve as an analog emulating how the screw would interact with the surrounding cortical wall, it has not been verified via mechanical comparisons between it and cadaveric models of a similar density. Therefore, future studies with this model should be performed in conjunction with cadaveric specimens and a large enough sample size to sufficiently increase the power of the study.

Final recommendations for future research can be made on the design of the expandable pedicle screw. In order to increase pullout strength, a wider arm or additional arms could be strategically incorporated to the screw in such a way that they maximize the surface area making contact with the cortical wall of the vertebral body while still expanding in specific locations (i.e., laterally and superiorly to the pedicle) to represent the least risk of neurological damage in case of failure. Moreover, an arm-retraction mechanism should be incorporated to avoid damage to the pedicle in the event that the screw needs to be removed after implantation. This was not included in the current design as the purpose of the study was to test the feasibility of a new design concept rather than testing a screw about to be used clinically. Adding such a mechanism at this stage would have significantly increased production time past what is desired at such an early phase in the product's development.

## 5. Conclusions

The single-armed expandable pedicle screw design could not outperform a standard pedicle screw in a pullout test performed in a synthetic model; however, the more gradual decrease in axial load following pullout observed with the expandable screw may potentially result in a higher level of stability as the patient waits for revision surgery in the case of instrumentation's failure. Furthermore, the expandable pedicle screw was successful in limiting damage to the desired location, offering a minimum risk of neural damage in the event of pullout. Damage to the cortical layer of the composite block revealed that the expandable pedicle screw may be as safe as, if not safer than, a standard pedicle screw instrumented in an osteoporotic vertebra.

## Figures and Tables

**Table 1:** Mean and standard deviation for all mechanical parameters recorded.

	Trabecular block				Trabecular block (with PMMA augmentation)				Composite block			
	Pullout (N)	Stiffness (N/mm)	Energy to failure (N∗mm)	Energy to removal (N∗mm)	Pullout (N)	Stiffness (N/mm)	Energy to failure (N∗mm)	Energy to removal (N∗mm)	Pullout (N)	Stiffness (N/mm)	Energy to failure (N∗mm)	Energy to removal (N∗mm)
Standard												
Mean	339	171	425	1300	338	161	499	2570	303	177	318	942
Standard deviation	9	7	21	21	69	12	208	229	10	25	61	133
Expandable												
Mean	321	160	396	1650	360	164	529	3200	293	179	336	1380
Standard deviation	10	11	33	68	28	6	86	527	31	21	94	199
